# Effect of variations in depth of neuromuscular blockade on rating of surgical conditions by surgeon and anesthesiologist in patients undergoing laparoscopic renal or prostatic surgery (BLISS trial): study protocol for a randomized controlled trial

**DOI:** 10.1186/1745-6215-14-63

**Published:** 2013-03-01

**Authors:** Martijn Boon, Christian H Martini, Leon PHJ Aarts, Rob FM Bevers, Albert Dahan

**Affiliations:** 1 Department of Anesthesiology, Leiden University Medical Center, P5-Q, RC Leiden, 2330, The Netherlands; 2 Department of Urology, Leiden University Medical Center, RC Leiden, 2300, The Netherlands

**Keywords:** Anesthesia, Neuromuscular block, Surgical rating scale

## Abstract

**Background:**

Surgical conditions in laparoscopic surgery are largely determined by the depth of neuromuscular relaxation. Especially in procedures that are confined to a narrow working field, such as retroperitoneal laparoscopic surgery, deep neuromuscular relaxation may be beneficial. Until recently, though, deep neuromuscular block (NMB) came at the expense of a variety of issues that conflicted with its use. However, with the introduction of sugammadex, rapid reversal of a deep NMB is feasible. In the current protocol, the association between the depth of NMB and rating of surgical conditions by the surgeon and anesthesiologist is studied.

**Methods/design:**

This is a single-center, prospective, randomized, blinded, parallel group and controlled trial. Eligible patients are randomly assigned to one of two groups: (1) deep NMB (post-tetanic count, one or two twitches; *n* = 12) and (2) moderate NMB (train-of-four, 1 to 2 twitches, *n* = 12) by administration of high-dose rocuronium in Group 1 and a combination of atracurium and mivacurium in Group 2. The NMB in Group 1 is reversed by 4 mg/kg sugammadex; the NMB in Group 2 by 1 mg neostigmine and 0.5 mg atropine. Patients are eligible if they are over 18 years, willing to sign the informed consent form, and are scheduled to undergo an elective laparoscopic renal procedure or laparoscopic prostatectomy. A single surgeon performs the surgeries and rates the surgical conditions on a five-point surgical rating scale (SRS) ranging from 1 (poor surgical conditions) to 5 (excellent surgical conditions). The intra-abdominal part of the surgeries is captured on video and a group of five anesthesiologists and ten surgical experts will rate the videos using the same SRS. The primary analysis will be an intention-to-treat analysis. Evaluation will include the association between the level of NMB and SRS, as obtained by the surgeon performing the procedure and the agreement between the scoring of the images by anesthesiologists and surgeons.

**Discussion:**

We aim to show that under the right conditions the perceived opposing goals of surgeons and anesthesiologists (optimal surgical conditions vs. optimal postoperative conditions) may be met without compromise to either.

**Trial registration:**

ClinicalTrials.gov identifier NCT01631149.

## Background

Surgical conditions in laparoscopic surgery are largely determined by the depth of neuromuscular relaxation during surgery. Especially in procedures that are confined to a narrow working field, such as retroperitoneal laparoscopic surgery (for example*,* renal and prostatic laparoscopic surgery), deep neuromuscular relaxation may be beneficial. Until recently, however, deep neuromuscular block (DNB, one or two twitches post-tetanic count (PTC)) came at the expense of a variety of issues that conflicted with its use. For example, occurrence of residual postoperative neuromuscular blockade after deep neuromuscular block (DNB) is a common adverse event (AE). Residual NMB is a risk factor for developing airway obstruction, hypoxia, and pulmonary complications, such pneumonia and atelectasis [1]. The use of acetycholinesterase inhibitors, such as neostigmine, to reverse NMB, while beneficial, is relatively slow in onset of reversal. Furthermore, side effects due to acetylcholine-induced muscarine receptor stimulation are bradycardia, hypersalivation, nausea, and vomiting [2]. Coadministration of a muscarine agonist, such as atropine or glycopyrrolate, is often necessary to counteract some of these side effects. These agents, however, in themselves may induce other side effects. Moreover, recent studies indicate that high-dose neostigmine (>5 mg) is associated with postoperative complications, including the need for reintubation and muscle weakness [3,4].

Rapid, complete reversal of neuromuscular blockade was not possible until the discovery of sugammadex. Sugammadex is a modified γ-cyclodextrin. It was developed to selectively bind free plasma rocuronium, a nondepolarizing steroidal neuromuscular blocking agent [5]. By binding free rocuronium, less rocuronium becomes available at the neuromuscular junction to bind to the muscarinic receptor [6]. Nondepolarizing neuromuscular blocking agents (NMBAs), such as rocuronium, block the muscarine receptors at the neuromuscular junction, making them unavailable to acetylcholine-based signal transmission. Sugammadex has proven to reverse rocuronium-induced NMB rapidly [7-10]. Even high-dose rocuronium (1.2 mg/kg) and continuous rocuronium infusion used to achieve deep neuromuscular blockade can be reversed by sugammadex [11,12].

Theoretically, the availability of sugammadex makes it possible to use DNB during surgery to improve surgical conditions without the occurrence of the above-mentioned AEs. The use of DNB to improve surgical conditions, however, has not yet been evaluated. This protocol was designed to study the relationship between the depth of NMB and the rating of the surgical conditions by the surgeon. To that end, either a deep NMB will be achieved with a rocuronium bolus administration followed by a continuous infusion (reaching 1–2 PTC) or a moderate NMB with a combination of an atracurium bolus administration and a mivacurium continuous infusion to obtain a level of NMB with a train-of-four (TOF) of 1 to 2. A combination of atracurium and mivacurium (two nondepolarizing NMBAs) is chosen, as they are part of the current anesthetic practice in our hospital for laparoscopic urological surgery. The deep block obtained with rocuronium will be reversed with sugammadex; the moderate block with neostigmine. During the laparoscopic procedure, the surgeon will rate the surgical conditions at 15 min intervals using a five-point surgical rating scale (SRS). To standardize the surgical rating, all surgeries are performed and rated by one surgeon with ample experience in the performed surgeries (RB).

A secondary aim of the study is to assess whether the anesthesiologist (the provider of the NMB and consequently responsible, in part, for the quality of the surgical conditions) is able to quantify the surgical field in surgical terms and also to assess whether the surgeon and anesthesiologist agree in perception or whether there is a disconnect in the opinions of surgical conditions between the two. This is important, as the request of surgeon for additional neuromuscular blockade is often not well received by the anesthesiologists (especially not at the end of the case) owing to concerns for residual NMB and other postoperative complications. To this end, video images of the intra-abdominal part of surgery are captured and rated (using the SRS) by five anesthesiologists and ten surgical experts, who serve as a control group.

Additional secondary endpoints of the study include the effect of depth of NMB on economic parameters (time to optimal extubation conditions (TOF 4 with T1/T4 ratio >90%), time to extubation, duration of surgery, and time in the post-anesthesia care unit), perioperative hemodynamics, and postoperative conditions (respiratory rate, arterial oxygen saturation, pain, sedation).

We hypothesize that:

1. There is a positive association between the depth of NMB and the surgical condition as rated by the attending surgeon.

2. There is a disconnect between the perception of the surgeon and the anesthesiologist in the rating of the surgical conditions.

3. Reversal of a deep NMB with sugammadex leads to postoperative conditions similar or superior to the conditions obtained from reversal of a moderate NMB with neostigmine.

## Methods and design

The protocol (P12.101) of the study was approved by the local ethics committee (Commissie Medische Ethiek, Leids Universitair Medisch Centrum) and registered at clinicaltrials.gov under number NCT01631149. The study is performed in one center, Leiden University Medical Center. All procedures will be executed in compliance with the current revision of the Declaration of Helsinki and Good Clinical Practice/Good Research Practice (GCP/GRP) guidelines. The study is randomized (deep versus moderate block) and blinded (the surgeon and the team that scores the videos are blinded; the attending anesthesiologist is not blinded).

### Inclusion and exclusion criteria

Patients diagnosed with renal or prostatic disease who will undergo elective laparoscopic renal surgery or a prostatectomy are eligible for inclusion in the study. Exclusion criteria are: American Society of Anesthesiologist class IV; age <18 years; inability to give informed oral or written consent; known or suspected neuromuscular disorders impairing neuromuscular function; allergies to muscle relaxants, anesthetics or narcotics; a (family) history of malignant hyperthermia; a contraindication for neostigmine administration; pregnancy or breastfeeding; renal insufficiency, as defined by serum creatinine levels at twice the normal level, or urine output <0.5 ml/(kg h) for at least 6 h (when available, other indices will also be taken into account, such as glomerular filtration rate <60 ml/h and proteinuria (a ratio of 30 mg albumin to 1 g of creatinine)), previous retroperitoneal surgery at the site of the current surgery, or body mass index >35 kg/m [2].

### Investigational plan and treatments

Eligible patients will be randomly assigned to one of two study groups. Group 1 will receive DNB (PTC of one or two twitches) obtained with a loading dose of rocuronium of 1.0 mg/kg followed by a continuous infusion. The pump rate will vary and depends on the PTC value. The initial pump rate will be set at 0.6 mg/kg per h. In case of a deviation from the required PTC value the pump rate can be increased or decreased. Group 2 will receive moderate NMB (TOF of one or two twitches) obtained with a loading dose of atracurium of 0.5 mg/kg followed by a continuous infusion of mivacurium. The pump rate will vary and depends on the TOF value. The initial pump rate will be set at 0.5 mg/kg per h. In case of a deviation from the required TOF value, the pump rate can be increased or decreased. In case of extreme poor conditions (SRS = 1 or 2), both rocuronium and mivacurium infusion rates will be increased by 20% after a bolus administration of 15 mg.

Since the research team is blinded to the muscle relaxant used and the level of NMB, the attending anesthesiologist is responsible for both the administration of the muscle relaxant and the degree of NMB. He or she will not discuss the NMB with the surgeon or the research team.

After the surgery has finished, subjects who receive rocuronium will be reversed with sugammadex (4 mg/kg). Again, this will be known by the attending anesthesiologist only and will be blinded to surgeon and the research team. Subjects who receive atracurium and mivacurium will be reversed with neostigmine (1 to 2 mg) and atropine (0.5 to 1 mg). Patients will be extubated when the TOF ratio becomes 0.9 or larger.

**Figure 1:**
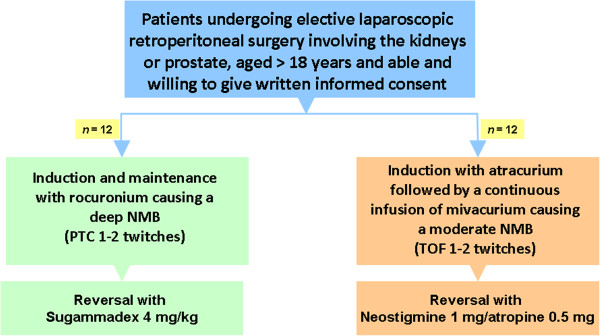
Study flow chart.

See Figure 1 for a flow diagram of the study.

### Anesthesia

Induction and maintenance of anesthesia is by propofol combined with bolus doses of an opioid (sufentanil). Propofol will be given as bolus of 2 to 2.5 mg/kg, followed by a continuous infusion of 8 to 12 mg/kg per h. The propofol dose will be adjusted such that the bispectral index (BIS, Covidien, Mansfield, MA) is between 45 and 55. The opioid use will be at the discretion of the attending anesthesiologist and is administered in single bolus infusions of 0.25 to 0.6 μg/kg. For NMB, see above.

### Study measurements during anesthesia

#### Surgical rating

During the laparoscopic procedure, the surgical condition is scored by the surgeon using a five-point SRS. To reduce variability in the surgical rating, all surgeries are performed by a single surgeon (RB). The rating scale will be a five-point ordinal scale ranging from 1 = poor condition to 5 = optimal surgical conditions (see Table 1). The surgeon scores the condition at 15-minute intervals. In case of a sudden change in surgical conditions, additional scores are obtained. If conditions are poor (score 1 or 2), muscle relaxation is increased according to protocol.

**Table 1:** Surgical rating score


1	Extremely poor conditions
2	Poor conditions
3	Acceptable conditions
4	Good conditions
5	Optimal conditions

Before commencing the study, both the surgeon and the anesthesiologists will receive training in how to use and implement the specific study procedures. Moreover, the first five procedures performed in conformance with this protocol will not be used for data analysis; thus, the procedures will be practiced five times before data collection starts.

#### Video images

The video images will be collected from the camera connected to the endoscopic probe placed in the retroperitoneal field of surgery. The images collected are the images used by the surgeon to observe the surgical field throughout the procedure (these are his working images). Thus, the collected video images are identical to the image of the surgeon during the procedure. The video images will be edited into short 30-s snippets at 15-min intervals (corresponding with the moment of surgical scoring) plus additional frames from episodes in which sudden changes in surgical conditions occurred. The videos will be rated by surgical experts (five urological surgeons with expertise in laparoscopic surgery, five non-urological surgeons with expertise in laparoscopic surgery) and five anesthesiologists (with expertise in giving anesthesia for laparoscopic procedures). These experts are blinded to the level of NMB and have no knowledge of the study endpoints. The rating score will be identical to the surgical rating score (Table 1).

#### Neuromuscular monitoring

For neuromuscular function monitoring, the TOF-watch SX acceleromyograph (MSD BV, Haarlem, The Netherlands) at the adductor pollicis muscle will be used. The thumb is attached to a flexible adaptor that applies a constant preload to the thumb. Neuromuscular stimulation will be applied to the ulnar nerve via two pediatric electrodes applied on the skin left and right of the ulnar nerve at the distal underarm. Before administration of any neuromuscular blocking agent, but after induction of general anesthesia, the following procedures are conducted to standardize the neuromuscular monitoring: (1) application of a tetanic ulnar nerve stimulation (50 Hz for 5 seconds); (2) calibration of the TOF-watch (CAL 2); (3) performance of a series of TOF measurements, to ensure that the TOF ratio differs by less than 5% between measurements (if the TOF ratio differs by more than 5%, the TOF-watch will be recalibrated). After these steps, the neuromuscular blocking agent according to protocol is administered. When, during the study, the number of measured twitches is zero, the PTC will be measured. A 50 Hz tetanic stimulation for 5 s is followed (after a 3 s rest) by 15 single stimulations at 1 Hz. The number of twitches generated (that is, the PTC) corresponds with the degree of NMB, with a PTC of 1 to 2 reflecting a deep level of NMB.

#### Hemodynamic monitoring

Blood pressure and cardiac output will be measured using the noninvasive NexFin device (Nexfin, Amsterdam, The Netherlands). This is a noninvasive beat-to-beat blood pressure measurement device and cardiac output monitor.

#### Intra-abdominal pressure

Intra-abdominal pressure will be measured every 15 minutes from the retroperitoneal CO_2_ insufflation device.

#### Additional variables

Additional variables will include duration of surgery, drug dosages (propofol, sufentanil, muscle relaxant, reversal agent, atropine, other agents used during anesthesia), duration from reversal to extubation, BIS, and ventilatory variables (tidal volume, respiratory rate, breathing pressure).

### Study measurements in the post-anesthesia care unit

In the recovery room, the following variables will be measured at 15 min intervals: respiratory rate, arterial oxygen saturation, numerical pain rating (on a scale from 0, no pain, to 10, most severe pain imaginable), occurrence of nausea or vomiting, and level of sedation or alertness. The level of sedation/alertness will be assessed in the recovery room using the validated Leiden Observer’s Assessment of Alertness/Sedation (Table 2) [13].

**Table 2:** The Leiden Observer’s Assessment of Alertness and Sedation scale


0	Normal alertness, eyes open, responds normal to command
1	Drowsy with open eyes, closed and opened on command
2	Drowsy with closed eyes, opened in response to light auditory stimulus
3	Eyes closed, opened in response to rubbing the shoulder or a load auditory stimulus
4	Eyes closed and opened only briefly in response to touching the subject
5	Eyes closed, not aroused by touching the subject, aroused by a painful stimulus
6	Not aroused by a painful stimulus

### Economic parameters

Time to optimal extubation conditions from the moment of reversal (with optimal conditions defined as TOF 4 with ratio >90%), time to extubation, duration of surgery, time in the post-anesthesia care unit, and drug consumption will be recorded.

### Safety evaluations

Adverse events (AE) that may occur during the study will be recorded in the case record form. The AE record in the case record form includes the nature of the event (with onset date and time, end date and time), severity, treatment, outcome, and the relationship to the treatment given. All serious AEs will be reported to the medical ethics committee and authorities, as required by the GCP/GRP guidelines.

### Sample size calculation

The sample size calculation is based on the expectations of the surgical rating score during surgery, as provided by an interview with the surgical expert (see Table 3).

**Table 3:** Approximate expectations of SRS for the two treatment arms

**Score**	**Interpretation**	**Treatment group**	
		**Moderate block**	**Deep block**
5	Optimal conditions	10%	70%
4	Good conditions	20%	20%
3	Acceptable conditions	55%	10%
2	Poor conditions	10%	0%
1	Extremely poor conditions	5%	0%

These anticipated frequencies lead to an odds ratio of 21 for obtaining optimal conditions versus suboptimal conditions for deep block as compared with the moderate block condition. To obtain the power for a given sample size, ten thousand simulations were performed with moderate block as a fixed distribution and a simulated distribution of the deep block condition, assuming proportionality of the odds ratio with an odds ratio of 21 and analyzing the results with a proportional odds model using the score test. Results of the simulations are given in Table 4.

**Table 4:** Approximate expectations for the two treatment arms

**Sample size per group (total)**	Power*
7 (14)	82%
8 (16)	87%
9 (18)	90%
10 (20)	93%
11 (22)	95%
12 (24)	97%

Given uncertainty of the anticipated effect size, a sample size of 12 per group or 24 in total was considered prudent, in order to provide a sufficient margin.

### Statistical analysis

The data analysis will be based on an intention-to-treat approach. For the SRS and other ordinal outcomes, the proportional odds model will be used, using the score test. If the proportionality of odds assumption is systematically violated, the data will be dichotomized for optimal conditions versus nonoptimal conditions. For continuous variables a general linear model will be used. Covariates can be included in these analyses when they provide an improvement of the model (when statistically significant at the 0.05 significance level, two-sided). Covariates to be considered are anesthesia or surgery related (for example, drug dosages, duration of surgery, BIS) and patient related (for example, age, weight, body mass index). Additional analyses will be performed on the secondary outcome parameters. To assess the agreement on the SRS across the various groups of raters the weighted kappa coefficient will be presented.

## Discussion

This is the first trial to assess the relationship between the level of neuromuscular blockade and the rating of surgical conditions in laparoscopic retroperitoneal surgery. To reduce between-subject variability in the scoring system, a single surgeon was selected to perform the procedure and score the surgical conditions. The surgeon in our study has ample experience with the procedure. We argue that this allows for sufficient confidence in the reliability of the SRS. The scoring scale was constructed in close cooperation between researchers and surgeon and based on a series of study try-outs. A five-point scale allows sufficient discrimination between surgical conditions varying from poor (SRS = 1) to excellent (SRS = 5). While it is expected that a DNB provides improved surgical conditions in laparoscopic retroperitoneal surgery, its use has been limited, owing to anticipated negative consequences, including the need for high-dose neostigmine for reversal, longer reversal times with consequently longer turn-over times, a high probability of poor postoperative conditions with the possibility of residual neuromuscular blockade and all of its consequences (reduced ability to fend hypoxia, aspiration, pneumonia, and so on) and prolonged stay in the post-anesthesia care unit [1,14]. Rapid reversal of a DNB is made possible with the introduction of sugammadex. However, rapid reversal is only acceptable when postoperative conditions are not different from those observed after moderate NMB and reversal with neostigmine and recurarization does not occur. Our protocol will shed light on this issue, as additional (secondary) aims include the assessment of respiration and pulse oximetry, as well as other patient conditions, in the recovery period.

An important part of our protocol is the study of the agreement between surgeon and anesthesiologists on the surgical conditions induced by the level of NMB. We do so by having a group of anesthesiologists score the surgical field using the video images obtained during surgery and rated by the surgeon. Since video images are dissimilar to the actual performance of the procedure, we will use a group of surgeons with expertise in laparoscopic surgery as a control group. In our experience, surgeons and anesthesiologists do not always agree on the optimal course of a procedure; this may be partly due to anesthesiologists not always being aware of the impact of anesthesia on the surgical conditions and surgeons not being aware of the impact of anesthesia on the postoperative course of the patient. Our study is aimed to show that under the right conditions the perceived opposing goals of surgeons and anesthesiologists (optimal surgical conditions vs. optimal postoperative conditions) may be met without compromise to one or the other.

## Trial status

Approval of the protocol has been obtained and recruitment of the patients is initiated. The study end is planned in May 2013.

## Abbreviations

AE: Adverse events; BIS: Bispectral index; DNB: Deep neuromuscular block; GCP/GRP: Good Clinical Practice/Good Research Practice; NMB: Neuromuscular block; NMBA: Neuromuscular blocking agent; PTC: Post-tetanic count; SRS: Surgical rating scale; TOF: Train-of-four.

## Competing interests

The authors declare that they have no competing interests.

## Authors’ contributions

AD and LA had the primary idea of the study. All authors contributed to the writing of the protocol. AD wrote this paper in close cooperation with MB and CM. The study will be executed by MB, CM, and AD. The surgical rating during the surgeries will be obtained from RB (urologist). Data analysis will be performed by CM and AD. All authors read and approved the final manuscript.
